# Engagement of networks related to attention, executive function, and sensory processing during parental vs experimenter story-listening: an fMRI study

**DOI:** 10.1038/s41390-025-04297-2

**Published:** 2025-08-04

**Authors:** Tzipi Horowitz-Kraus, Liana Magaliff, Dror Kraus, Mika Shapira Rootman, Tamar Steinberg, Dorit Aram, Rupa Radhakrishnan, Rola Farah

**Affiliations:** 1 Educational Neuroimaging Group, Faculty of Education in Science and Technology, Technion- Israel Institute of Technology, Haifa, Israel; 2 Faculty of Biomedical Engineering, Technion- Israel Institute of Technology, Haifa, Israel; 3 TecHMRC, Technion Human MRI Research Center, Technion- Israel Institute of Technology, Haifa, Israel; 4 Department of Neuropsychology, Center for Neurodevelopmental and Imaging Research (CNIR), Kennedy Krieger Institute, Baltimore, MD USA; 5 The Institute of Child Neurology SCMCI, Petach-Tikva, Israel; 6 Faculty of Medicine, Tel-Aviv University (TAU), Tel Aviv, Israel; 7 Department of Radiology, Rambam Hospital Medical Center, Haifa, Israel; 8 Faculty of Medicine, Technion - Israel Institute of Technology, Haifa, Israel; 9 School of Education, Tel-Aviv University (TAU), Tel Aviv, Israel; 10 Department of Radiology, School of Medicine, Indiana University, Pennsylvania, IN USA

## Abstract

**Background:**

Caregivers shape children’s reading abilities and neural networks. While joint reading is well-studied, the impact of parental storytelling on reading-related networks (executive function [EF], attention, sensory processing) before and at the early stages of reading acquisition remains unclear. This study examines whether parental storytelling differentially influences these networks compared to an experimenter’s narration.

**Methods:**

Twenty-four Hebrew-speaking children (5.0–7.11 years; six females) and their parents participated. Pre-literacy and cognitive skills were assessed, and parents completed cognitive control and language questionnaires. Functional MRI data were collected as children listened to their parent narrate a picture-book story and an experimenter read an age-matched story. Functional connectivity within and between attention, EF, language, and sensory networks was analyzed and correlated with behavioral measures.

**Results:**

Parental storytelling elicited stronger connectivity across all networks. Increased EF, attention, and sensory network connectivity correlated positively with book exposure and negatively with reading hours. Stronger sensory processing connectivity during parental narration was linked to better pre-literacy skills. Enhanced audiovisual integration correlated with home literacy, pre-literacy, and later reading abilities.

**Conclusions:**

Parental storytelling engages reading-related networks, supporting pre-literacy and future reading skills beyond exposure to an unfamiliar reader.

**Impact:**

- The literature in the field of parent-child interaction and language development repeatedly points to the benefit of book reading to the child with respect to language development.
- However, whether there is a particular benefit specifically for parental reading is unknown and whether the child’s brain is stimulated differently when the parent is reading a book (vs an experimenter) is also unknown.
- Our results show that parental reading engages brain regions related to cognitive abilities, attention, language abilities and future reading skills in children even prior to reading age. Parental reading is important for the development of these brain regions.

## Introduction

### The effect of joint storytelling with the child on the child’s language, cognitive and emotional development

One of the best ways to engage with a child on emotional as well as cognitive levels is through reading together.^1,2^ Joint parent-child reading builds attachment and is also key for expanding linguistic skills, emergent literacy and promoting later success in independent reading.^1,3^ There are several techniques for storytelling with the child: dialogic reading (DR), shared reading, and storytelling to the child. Reading with children should begin even before the child can speak. The American Academy of Pediatrics recommends using a shared or DR technique as early and as often as possible, as reading aloud together benefits the child’s brain from birth.^4,5^ The nonprofit Reach Out and Read group incorporates developmentally appropriate storytelling and literacy into a child’s healthcare plan from the age of six months, encouraging joint attention and parental bonding.^6^ Engaging in reading conversations even before language ability fully develops enhances the child’s vocabulary, verbal engagement, sensory associations, and literacy skills, as well as their connection with the caregiver.^4^ Further benefits of DR and shared reading include enhancing expressive language, narrative comprehension, and attention—skills necessary for emergent literacy.^1^ Is storytelling different when narrated by the child’s parent or caregiver compared to a stranger? This study aims to examine this question in depth.

### Greater attention and visualization are present during parental reading

Specific benefits of parental reading include enhancing oral or expressive language, embracing literacy seeds (or emergent literacy, which is the understanding that written materials have a meaning in spoken language, directionality of the writing system, the concepts of letters, words and sentences^7^), an overall increase in basic and health literacy, and closing the word gap.^1,3^ The word gap is a term used to describe the notion that children from lower socioeconomic status (SES) backgrounds are exposed to fewer spoken words. It is suggested that the word gap can be remedied, meaning that children from lower SES backgrounds can learn the same number and level of words as their higher-SES peers through interventions such as shared reading.^8^ This joint parental reading activity will help promote future literacy readiness for all children, regardless of their background,^8^ by teaching them more words at an appropriate developmental level.

Reading, however, is relatively new within the timeframe of human evolution, invented around 5000 years ago.^8^ Therefore, it is worth exploring the consequences of this activity within different dynamics of learning, including parent-child storytelling, to help us understand its evolution and cultivation. Studies show that attention is increased during and as a result of joint reading.^2^ It was previously suggested that increased attentiveness and visualization, more exploratory behaviors, and better self-regulatory skills during joint reading are, in part, positively correlated to the emotional attachment the child experiences when spending time reading with a parent, as compared with an experimenter.^1,2,9^ This emotional attachment highlights the necessity of safety, security, and bonding within the caregiver-child relationship to increase emotional and social development.^10^

Narrative comprehension is usually measured behaviorally using pencil and paper tests, only after the child listens to the story, while neuroimaging and physiological data can provide mechanistic insights regarding special characteristics of parental storytelling while examining brain activation or functional connection during the storytelling activity. Zivan and colleagues, for example, were using an eye-tracker device to demonstrate different eye-movement exploration patterns that 4–6-year-old children used when the parent read them a story vs an experimenter [2]. In that study, a greater exploration patterns manifested by more and longer fixations on letters (compared with pictures) among pre- and early readers. Since the interaction with the parent is the first interaction in the child’s life, and as storytelling is also related to bonding, different pitch and prosody,^11,12^ it is of interest to explore if there is a specific neurobiological signature specific for parental reading, in relations to environmental factors such as the home literacy environment.

### Factors affecting narrative comprehension: home literacy environment (HLE) and parent-child interaction

The quality of the joint parental reading activity is crucial, determined in part by the level of home literacy environment (HLE), SES, and child’s involvement.^1,3,13^ A high-quality experience of joint reading with a parent includes measures of “verbal interactivity and engagement”.^1^ This includes child-directed speech, dialogic or conversational reading,^3^ and behaviors of the reader that provide assistance when necessary, allowing the child to actively engage without distraction or control from the narrator.^1,9^ This high level of parent-child engagement was associated with greater activation of brain regions related to learning and future reading skills, such as the cerebellum and left angular and insular gyri.^14^

Preceding opportunities for joint parent-child reading is the necessity for the child to feel secure, hold attention, understand the story’s context via listening and visualization, and ultimately emit a level of cognitive control or EF that allows for processing all aspects of the activity. While these innate skills are forming, the child is simultaneously building networks required for emergent literacy, referred to as “reading-related networks”, which characteristics during parental vs experimenter storytelling we aim to explore through our research.

### Story-listening relies on abilities supporting future reading: language, attention, visualization, and EF

The ability to listen to stories relies on more basic cognitive abilities, such as attention, visualization or mental imagery of the story, EF, and, through learning of oral language, narrative comprehension and reciprocal conversation with the narrator.^3,13^ As visual and oral language processing precede semantic processing, language acquisition is necessary for passive story-listening.^3,13^ Younger children have greater neuroplasticity; thus, it is ideal to build these networks early,^13^ beginning with joint parental reading. Shared reading quality, such as DR and reciprocal interaction, can also be a predictor of neural activation,^1^ and though child participation helps, parental joint reading with only verbal stimulation still benefits the child’s neural development.^1^ Executive functions mature later and support the development of cognitive and emotional processing abilities.^3^ Neuroimaging techniques, including fMRI and EEG, show that the brain regions related to EF are activated during tasks of passive story listening and narrative comprehension, skills which later enhance the child’s ability to read.^3^ Selected regions in the brains of preschoolers are activated upon exposure to joint reading and time spent with parents.^1,2^ The left parietal, temporal, and occipital (PTO) association cortex and white matter myelination support improvement in semantic processing, somatic sensations, attention, and language skills.^1,2^ While completing a story listening task within a stimulating home environment offering books, toys, and joint reading opportunities,^3,13^ the brain regions involved in visual-semantic processing, vocabulary learning (left angular gyrus), and EF were associated with the child’s emergent literacy skills later.^2,3,13^ Increased scores of home reading exposure were positively correlated with left PTO cortex activation,^13,14^ suggesting that a stimulating home environment helps to build networks supporting mental imagery and narrative comprehension.^13,14^ Mental imagery (or visualization) describes the process of creating images or scenes in one’s head even without external sensory stimulus.^15,16^ This visualization process is important for stimulating brain regions related to word reading within the reading network, even prior to reading age.^13,17^ Moreover, children with increased receptive vocabulary demonstrated a greater left lateralization, specifically activation in the angular and supramarginal gyri as well as the thalamus, which play a part in semantic processing.^2,18^ This left lateralization, especially in reading-related regions, is important for future reading skills.^19^ Reading, one of the most challenging and non-intuitive human abilities of translating letters into sounds and spoken language, shares several cognitive abilities with story-listening.^20^ The neural circuits supporting semantic processing are required for narrative comprehension and later reactivated to aid in reading comprehension.^20^ Studies examining the relations between pre-readers listening to stories and future reading outcomes revealed that the utilization of frontal and supramarginal gyri, temporal-occipital activation, EF, and sensory regions while listening to stories at age five years (prior to reading age) are related to greater reading outcomes at age 11 years.^20^ The authors suggested that the ability to imagine the stories and visualize them while listening is related to the ability to recognize words during reading age.

However, as parental storytelling is critical, as explained above, it is still unknown if specific neural circuits are associated with a parental storytelling vs. an experimenter.

It is also not clear how much a synchronization of reading-related networks supporting reading, such as attention, EF, and the sensory system—the visual (supporting visualization) and auditory (supporting language) networks- during parental reading is related to the child’s reading readiness (i.e., literacy seeds).

Therefore, the goal of the current study is to determine the utilization of the above-mentioned reading-related networks– during parental storytelling and their relation to pre-literacy and future reading skills among a cohort of pre- and early-readers. We hypothesize that: 1) children will show better narrative comprehension scores (measured by a questionnaire) for the parental storytelling condition compared to the experimenter storytelling condition; 2) we also suggested that FC of children’s listening to parental storytelling, will be stronger compared FC of the reading-related network [attention and EF networks [Cingulo-Opercular (CO), Fronto-Parietal (FP)], attention networks [Ventral attention (VAN) and Dorsal attention network (DAN)] as well as sensory networks (auditory and visual networks)] for the experimenter condition; 3) increased HLE scores will be associated with increased FC in sensory networks and EF regions in the parent condition; and 4) increased FC between EF and attention networks during the parent condition will be related to better pre-literacy measures in the child. Finally, 5) we suggested that a greater engagement of reading-related networks (EF and sensory networks) during parental vs experimenter conditions will be related to better reading scores at reading age.

## Methods

### Participants

This study included dyads of mothers and their 5–7 years old children, enrolled in kindergarten to second grade. Inclusion criteria for this study were children at pre- and early-reading phase (*n* = 24, six females), born at term gestation, all from a native Hebrew-speaking household. Exclusion criteria were a history of neurodevelopmental disorders and contraindications to participate in an MRI scan. Mean maternal education was 17.17 ± 2.29 years (a minimum Bachelor’s degree), and an average SES (mean 15,000–25,000 NIS, minimum 10,000, maximum 35,000 NIS, 4 NIS = ~$1, reflecting a range of diverse SES). The Conners Rating Scale^21^ was used to assess the probability that a child has an attention disorder. Advertisements were used for child recruitment (such as on social media or presented at school). Two years after the first visit, reading measures were collected. Families were compensated for time and travel expenses. This study was approved by the Ministry of Health.

### Study Procedure

Parents and children were invited to the laboratory and were explained the procedures and instructions. After the parents signed the consent, behavioral testing began, followed by neuroimaging testing. The entire visit lasted up to three hours.

### Behavioral measures

The following set of questionnaires and tests was administered to assess participants’ behavioral and cognitive abilities:

#### General verbal and nonverbal abilities

To ensure an average nonverbal ability, the Wechsler Preschool and Primary Scale of Intelligence’s WPPSI matrix reasoning test for nonverbal intelligence was administered,^22^ and verbal ability was determined using the expressive vocabulary test.^22^

Cognitive Measures:

#### Executive function

Parental questionnaires regarding their children’s EF abilities were filled out (Behavior Rating Inventory of Executive Function (BRIEF-2).^23^

#### Linguistic measures

Semantic and verbal fluency were measured using subtests from the Shatil battery (including the naming objects and letters subtests^24^).

#### Pre-reading skills (phonological processing)

Phonemic awareness was measured using the phonemic awareness task from the Shatil battery.^24^

#### Home literacy environment (HLE)

The Stim Q-Preschool questionnaire^25^ was used to evaluate home literacy environment via the amount and frequency of literary visits number of books in the child’s home, number of hours read to the child, and play stimulation provided to the child within the home.

#### Reading Abilities

Reading measures (two years after the first testing) included the accuracy and speed for the contextual reading task (with punctuation) from the Aleph-Taph battery to assess reading fluency.^26^ Additional information on each test battery and the tests’ reliability is provided in the Supplemental Material.

### Neuroimaging measures

#### fMRI tasks

Children watched and listened to two an overall two prerecorded stories: The book “Should I Share my Ice Cream?” by Mo Willems (translated into Hebrew) was read out loud by the parent. The story told by the experimenter (“A Pigeon Finds a Hot Dog!” by Mo Willems, translated into Hebrew) was read out loud by a lab coordinator, prerecorded and consistent for all participants. The order of the books was randomized during the scan.

**Fig. 1:**
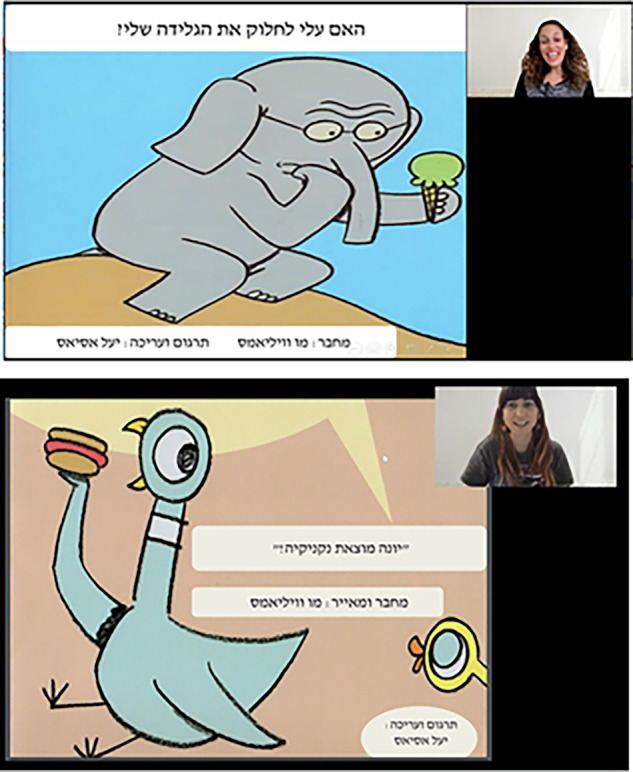
An example of screenshots of the two task conditions. Screenshots of the story-listening tasks told by the parent (Upper: “Ice Cream”), and by an experimenter (Lower: “Pigeon”).

The two stories were matched in author, illustration, difficulty, length, the main topic of the book (the theme focused on “sharing”), with a main character who was the focus, and an additional sub-character. These factors ensured that observed changes in functional connectivity could be attributed to our variable of interest (narrator) rather than the stories’ contents, making the data more reliable and interpretable. The books were selected by an expert on child literacy. During each scan, the child observed a video recording of the story, which also included the parental video (in the parental condition) and the experimenter (in the experimenter condition) to allow an exposure to the facial expression of the narrator (to get as close as possible to a real-life storytelling). The pages of the book could be seen on the main screen with the narrators in the upper right corner (see Fig. 1).

After the scan, participants answered five multiple-choice comprehension questions on each of the two stories’ contents (parent, experimenter).

For the production of the fMRI tasks, the books were scanned with each page appearing on a separate slide. The parents were provided a PowerPoint of the book and requested to use a Zoom recording while displaying the book on a full screen, allowing for the parent to be seen on the top right of the screen, narrating the story to the child as they would at home. The experimenter video was recorded similarly. After receiving each video, the video was formatted to fit the MRI computer screen’s dimensions at a resolution of 1920 × 1810 pixels. Each video was recorded for exactly 5 min.

#### Neuroimaging parameters

Neuroimaging measures included an anatomical scan (T1) and two additional functional (T2*-weighted) MRI paradigms: a) parental storytelling; and b) experimenter storytelling. Stimuli were presented via E-Prime 3.0 stimulus presentation for behavioral research.^27,28^ Functional MRI scans were performed using a 3T Siemens Prisma MRI (Siemens Healthineers, Erlangden, Germany) with an audiovisual feature (Psychology Software Tools, Pittsburgh, Pennsylvania) and a 64-channel head-neck coil to acquire neuroimaging data. Anatomical images were acquired using a T1-weighted MPRAGE pulse sequence parcellated into 1 × 1 × 1 mm^3^ voxels. Functional images were acquired using a T2*-weighted echo-planar imaging (EPI) pulse sequence with the following parameters: a multiband factor of 4, TR/TE = 1000/30 msec, flip angle = 68°, and voxel size 2 × 2 × 2 mm^3^. Each volume was comprised of 306 frames, each 2 mm thick.

A thorough desensitization process was used to acclimate the children to the machine, including sharing files with the scanner sounds, practicing staying still like a statue, as well as a test run in a mock MRI during the subsequent study. If overt movement was noted during the scan, the participants were asked to try to stay as still as possible, and the protocol continued.^29^

### Data analyses

#### Behavioral data analysis

Independent *t*-tests were conducted to determine the participants’ cognitive and linguistic abilities (means and standard deviations).

Paired *t*-tests were conducted to compare the comprehension scores outside the scanner in the two storytelling conditions.

#### Neuroimaging data analysis

Neuroimaging data were analyzed using CONN,^30^ SPSS,^31^ and Matlab^32^ softwares to gather information about FC during the tasks.

##### Data preprocessing

**Fig. 2:**
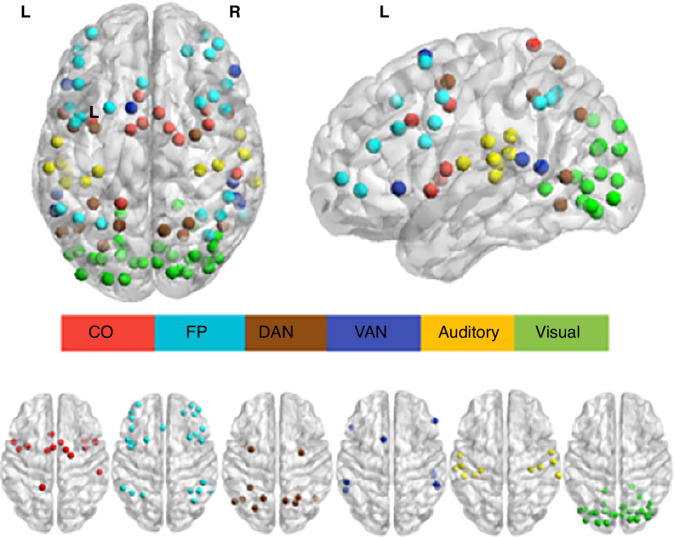
Functional connectivity networks corresponding to executive function, attention, and sensory processing. Functional networks masks based on the Power’s Atlas,^33^ plotted via the MNI space template coordinates using BrainNet Viewer.^39,40^ CO (ROIs 36–49), FP (ROIs 157–181), DAN (ROIs 222–232), VAN (213–221), Auditory (ROIs 50–62), and Visual (ROIs 126–156). L/R  left/right.

Functional MRI data was run through a preprocessing pipeline using CONN.^30^ Preprocessing included realignment using three translational and three rotational parameters, coregistration of the anatomical image to the mean aligned functional image, segmentation of the tissue types, normalization to the Montreal Neurological Institute-152 template, and spatial smoothing (kernel of 8 mm). The slice timing correction controlled for the effect of subject motion. Frames with movement >0.2 mm were excluded from the analyses. Following the cleaning and removal of noisy frames, first-level analysis was conducted to create ROI-to-ROI matrices per participant. This was done by applying the Power’s brain atlas,^33^ which comprises 264 ROIs across the entire brain, further forming 14 functional networks. The reading-related networks included EF networks (the cingulo-opercular; CO, fronto-parietal; FP), attention (dorsal attention, ventral attention; DAN, VAN), and sensory processing (auditory and visual networks). Within and between FC networks were defined and exported (see Fig. 2 for a spatial maps of the selected networks).

##### Comparisons of functional connectivity between parent and experimenter conditions

First, FC averages for each within-network and between-network connectivity were calculated for each network for each condition. Paired *t*-tests for FC averages within- and between-networks for each of the six networks separately and between-networks for each condition for a within-subjects analysis.

##### Correlations between functional connectivity and behavioral measures

Pearson correlations were conducted to correlate HLE scores, literacy seed and later reading abilities with FC means of the functional networks when the parent vs in the experimenter storytelling conditions.

## Results

### Behavioral measures

Children demonstrated average EF, attention, and language scores within the normal ranges (see Table 1).

**Table 1:** Descriptive statistics for the administered child behavioral battery.

Time of Testing	Ability	Test	Mean (SD)	Min–Max	Normal range	Confidence Interval (95%)
Pre-reading acquisition testing (mean age 77.88 ± 10.67 months), *n* = 24	Executive Functions	BRIEF GEC T score	43.55 (6.77)	34.00–60.00	40–59	41.76–48.45
	Language	Shatil Naming Standard Score (performance time)	0.21 (1.82)	−3.00–3.00	−1.50–1.50	−0.287–1.126
	Phonemic Awareness	Shatil Phonemic Awareness (Standard Score)	0.59 (1.58)	−2.00–3.00	−1.50–1.50	0.31.3–1.54
	Literacy Seed	Shatil Standard Score (based on accuracy)	0.77 (1.25)	−2.00–3.00	−1.50–1.50	0.4–1.313
	Literacy Seed (Language)	WPPSI Vocabulary Standard Score	9.91 (2.90)	5.00–16.00	7.00–13.00	8.99–11.2
	Home literacy environment	HLE, number of books	189.47 (130.70)	30.00–500.00	n/a	127.52–260.04
		HLE, weekly hours reading	2.92 (4.07)	0.00–20.00	n/a	1.25–4.48
Reading age testing (mean 104.34 ± 11.16 months), *n* = 20	Contextual Reading Speed	Reading for Context, punctuated (based on accurate WPM SD)	−0.16 (0.70)	−1.58–0.93	−1.50–1.50	−0.61–(−0.05)
	Contextual Reading Accuracy	Reading for Context, punctuated (based on percent mistakes SD)	−0.34 (0.60)	−1.97–0.36	−1.50–1.50	−0.48–0.17

*BRIEF GEC* Behavioral Rating Inventory of Executive Function, Global Executive Composite, *WPPSI* Wechsler Preschool and Primary Scale of Intelligence, *HLE* Home Literacy Environment.

### Neuroimaging measures

#### Narrative comprehension of the parent vs experimenter storytelling conditions

No significant differences in accuracy rate for narrative comprehension between the parent and experimenter storytelling tasks were found. See Table 2.

**Table 2:** Paired t-test comparing the narrative comprehension scores for parental vs experimenter storytelling conditions.

	Parent	Experimenter	*T*(p)	Confidence Interval (95%)
	Mean (SD) *n* = 24	Mean (SD) *n* = 24		
Narrative comprehension score (percent correct responses)	75.45 (25.66)	74.55 (21.41)	0.139 (0.445)	67.61-85.71

### Neuroimaging data

#### Network FC differences in the parent vs experimenter conditions

Significantly greater FC in the parent vs the experimenter conditions within- and between-networks were found for the EF networks (FP, CO-FP), between the EF and sensory (visual; FP-Visual networks) networks and the EF and attention networks (CO-DAN, FP-DAN, FP-VAN). See Table 3.

**Table 3:** Paired-sample *t*-tests of mean FC values within- and between-networks for both parent and experimenter storytelling conditions.

		Parent (*A*)	Experimenter (*B*)	*T*(p)	Contrast	Confidence Interval (95%)
		Mean (SD) *N* = 24	Mean (SD) *N* = 24			
Within-network FC	CO	0.249 (0.075)	0.221 (0.103)	1.544 (0.68)	*A* > *B*	−0.009–0.006
	FP	0.220 (0.055)	0.181 (0.065)	2.390 (0.013)*	*A* > *B*	0.005–0.073
	DAN	0.322 (0.085)	0.286 (0.113)	1.211 (0.119)	*A* > *B*	0.035–0.098
	VAN	0.327 (0.088)	0.275 (0.124)	1.700 (0.051)	*A* > *B*	0.24–0.33
	Auditory	0.362 (0.101)	0.352 (0.162)	0.374 (.356)	*A* > *B*	−0.04–0.06
	Visual	0.304 (0.077)	0.278 (0.097)	0.867 (0.198)	*A* > *B*	−0.03–0.86
Between-network FC	CO-FP	0.036 (0.065)	0.010 (0.049)	1.977 (0.030)*	*A* > *B*	−0.001–0.052
	CO-DAN	0.030 (0.070)	−0.005 (0.069)	2.292 (0.016)*	*A* > *B*	0.003–0.06
	CO-VAN	0.099 (0.072)	0.070 (0.067)	1.621 (0.059)	*A* > *B*	−0.007–0.06
	CO-Auditory	0.219 (0.065)	0.201 (0.098)	0.955 (0.175)	*A* > *B*	−0.021–0.05
	CO-Visual	0.024 (0.045)	0.004 (0.058)	1.627 (0.059)	*A* > *B*	−0.005–0.04
	FP-DAN	0.112 (0.067)	0.077 (0.052)	2.296 (0.016)*	*A* > *B*	0.003–0.06
	FP-VAN	0.072 (0.087)	0.039 (0.050)	1.761 (0.046)*	*A* > *B*	−0.005–0.07
	FP-Auditory	0.014 (0.080)	−0.010 (0.073)	1.228 (0.116)	*A* > *B*	−0.016–0.06
	FP-Visual	0.018 (0.055)	−0.011 (0.059)	2.016 (0.028)*	*A* > *B*	−0.007–0.059
	DAN-VAN	0.050 (0.078)	0.043 (0.073)	0.395 (0.348)	*A* > *B*	−0.029–0.04
	DAN-Aud	0.020 (0.071)	−0.004 (0.072)	1.336 (0.097)	*A* > *B*	−0.013–0.06
	DAN-Visual	0.165 (0.079)	0.136 (0.077)	1.558 (0.066)	*A* > *B*	−0.009–0.06
	VAN-Auditory	0.183 (0.084)	0.151 (0.088)	1.245 (0.113)	*A* > *B*	−0.021–0.085
	VAN-Visual	0.031 (0.085)	0.025 (0.060)	0.332 (0.371)	*A* > *B*	−0.03–0.042
	Auditory–Visual	0.032 (0.067)	0.013 (0.063)	1.330 (0.098)	*A* > *B*	−0.01–0.047

*CO* cingulo-opercular, *FP* fronto-parietal, *DAN* dorsal attention network, *VAN* ventral attention network.

*statistically significant at the *p* <  0.05 level.

## Discussion

The goal of this study was to determine the neural correlates involved in parental storytelling and their relation to future reading-related abilities such as EF, language, and HLE, in pre- and early-reading children. In line with our hypotheses, increased FC within EF networks and between EF-attention and EF-sensory networks were found when parents told the story vs the experimenter. Additionally, an increased HLE was associated with increased FC in networks that include regions associated with language processing and semantic processing regions (at least one network of interest per EF, attention, and sensory modalities). Finally, greater FC within and between reading-related networks was correlated with better reading performance 2 years later. Interestingly, (and as opposed to our original hypothesis), narrative comprehension scores did not differ between the two-story conditions. Increased FC within the sensory networks, and not the EF and attention networks, was related to better pre-literacy measures of the child. The following will be discussed below.

### A greater engagement of EF networks during parental reading

The FP network was previously related to higher-order cognitive skills, including but not limited to planning, problem-solving, and inhibition and is considered an EF network.^34^ Previous research suggested that joint parent-child reading fosters attachment and contributes to language abilities, literacy, and reading ability, all rely on EF.^9,11^ The foundational skills necessary for more complex abilities, including attention and visualization, are developed through the parent-child reading experience via the FP network, among others.

Negative functional connectivity among EF, attention, and sensory networks was found only during the experimenter storytelling condition, particularly between the CO-DAN and FP-visual processing correlation, where the differences in these networks’ interactions between the conditions were significant. This pattern suggests less efficient coordination across the systems, potentially reflecting attentional conflict or increased cognitive load in response to the experimenter telling a story. This aligns with the hypothesis of greater FC during parental storytelling, which may stem from the child’s attuned and established connection with their parent, allowing for a greater neural synchrony.^35^ The parent-child bond likely supports more synchronized and efficient activity, as engagement, attachment, and voice familiarity help reduce cognitive load and enhance integration across these systems (for support from an EEG data see ref. ^36^).

While narrative comprehension (measured by a questionnaire administered after the scan) did not significantly differ based on the narrator’s difference between the parental and experimenter conditions, our results provide neurobiological evidence of functional changes for both conditions. With this advantage of neuroimaging data, we can see critical networks more engaged with the parent storytelling condition which may represent the lower level of cognitive effort required in order to reach similar comprehension levels when the story is told by an experimenter. These findings are in line with previous eye tracking results characterizing prenatal vs experimental storytelling in 4–6 years old children.^37^

### Higher home literacy environment was related to greater functional connectivity of the components of the reading network (EF, attention, and sensory networks) in the parent vs experimenter storytelling condition

Home literacy environment is a crucial factor in shaping children’s experiences with learning, literature, and language processing, ultimately influencing academic achievement and both expressive and receptive language abilities.^38^ Parents (or primary caregivers) are instrumental in nurturing their children’s emergent literacy abilities and building brain networks that are later involved in fluent reading, including structures within the FP, DAN, and VAN.

Our results indicate that exposure to more resources at home (measured by book count) is positively associated with greater engagement of attention-sensory networks and alertness, aligning with previous findings. Another component of HLE, hours spent reading together (with a parent, as most of our participants could not yet read independently), was associated with less engagement in EF-sensory (FP-auditory) networks. This finding remained robust after excluding an outlier, strengthening the notion that shared reading time with a parent might reduce the cognitive load and demand on sensory processing during these early states of literacy development, as the effort the child experiences might be lower when processing the same information than if with an experimenter.

With these additional data, the results of the current study suggest that a higher-quality HLE contributes to a child’s developing reading network, supporting crucial attention and sensory processing skills for later reading.

### Greater literacy seeds were related to greater neurobiological correlates for visualization and sensory integration during parental storytelling

Literacy seeds refer to early signs or actions nurturing literacy development; to measure emergent literacy a bit later, we assess children’s early indications of understanding the reading process – concepts of print, phonological awareness, and letter recognition, among other elements. Like HLE, literacy seed actions can be fostered from birth and within the home, exposing children to basic concepts of reading and language.

Given that higher parental involvement increases the likelihood of children’s understanding of reading concepts, we expected that children would be more in tune with the narration from their parent compared with an experimenter. This is shown in our results of greater FC within and between sensory networks (auditory–visual) during parental storytelling. Again, a stronger bond with the parent can lend to a greater cognitive engagement.

### Greater FC during parental storytelling correlates with better future reading abilities, less comprehension

A parent is the child’s first introduction to reading and literacy exposure, which has a profound effect on their future ability, reading fluency, and academic achievement.^1,2,14^ The results of the correlation for reading abilities 2 years after the scans suggest that greater FC within- and between- our networks of interest during parental storytelling as compared with experimenter storytelling is associated with better long-term performance in reading ability, specifically in correct pronunciation of words and less variability in mistakes while reading a text. This implies there is a positive longitudinal effect of parental storytelling in children’s achievement of learning to read fluently.

Taken together, our results show greater FC during parental storytelling, compared with an experimenter narrating, and significant correlations with measures of narrative comprehension, literacy seeds, HLE, and later reading abilities. Most notably, only enhanced sensory processing (i.e., increased FC between the auditory and visual networks), reflecting the efficiency of audiovisual integration (AVI), in the parent storytelling condition, correlates with pre-literacy, HLE, and future reading. These results may point to the impact and benefit of enhanced sensory processing on the child’s reading-related network, during parental storytelling.

## Limitations

The significant results of the current study should be considered in light of the following limitations. Though the order of the fMRI tasks was randomized, the fMRI conditions in the current study were not randomized, such that sometimes the parents would read the Pigeon and sometimes the experimenter read the Elephant. Future studies should provide half the parents with one story and half with the other, as well as having the experimenter record themselves reading both. Moreover, although one of the exclusion criteria for the current study was a diagnosis of a neurodevelopmental disorder, the behavioral battery scores demonstrate a wide range of abilities. This may point to the variability of our participants, which suggests that the results can be generalized; however, it may also suggest that the participants in this study may include children who can potentially be diagnosed in the future as having learning difficulties. As the goal of the current study was not to examine precursors for learning difficulties, future longitudinal studies are warranted to address this question. In relation to the variability in behavioral scores, narrative comprehension level also differed between participants (range: 50–100% accuracy rate). This might be due to the range of cognitive ability mentioned earlier, or alternatively, due to the unnatural setting of storytelling in the scanner. Additional study examining comprehension level after a scanner-based storytelling vs outside of the scanner storytelling can address this point.

Another important point is that the current study does not include a reading task during the first study visit, to evaluate the participants’ baseline reading ability. Lastly, this study includes more male than female participants. While controlling for sex in our analyses, a main effect of sex was found for the CO-FP network [F(1, 22) = 8.51, *p* = 0.008, partial *η*²=0.279], suggesting that sex may have an effect on the differences in CO-FP engagement during parental vs experimenter conditions. Moreover, the correlations between future reading and visual and DAN-VAN were trending significance [Visual [*r* = −0.44, *p* = 0.057] and DAN-VAN [*r* = −0.41, *p* = 0.072]. These results point to sex a possible confounder of the results and point to a possible difference between females and males in processing stories told by a parent vs an experimenter. A future study examining the difference in sex of the participants as well as their parents (mothers vs fathers) can explore this point in depth.

### Future direction

The experimenter condition in the current study raises an interesting question regarding the role of others in the child’s life, such as their kindergarten teacher or the comparison between the father’s and the mother’s storytelling; it will be insightful to consider the impact of storytelling by a trusted adult who is further within the child’s extended circle – more familiar than a stranger but more distant than the parent. An examination of the neurobiological correlates in response to their teacher, with whom children (especially of reading age) spend many hours learning from and reading together, would be of interest. Moreover, expanding the current study’s results analytically, by using additional FC analysis methods such as multivariate analysis or alternatively, using graph theory measures, can also determine whether there are main hubs participating in the parental storytelling condition.

To enhance our understanding over a wider range of development, we can examine the functional data to determine the timing of left lateralization of reading. Along with measuring FC and behavioral abilities in various age groups and clinical populations, we will help to track reading and reading network development across childhood or the lifespan and examine its relationship within various clinical presentations.

## Conclusions

**Fig. 3:**
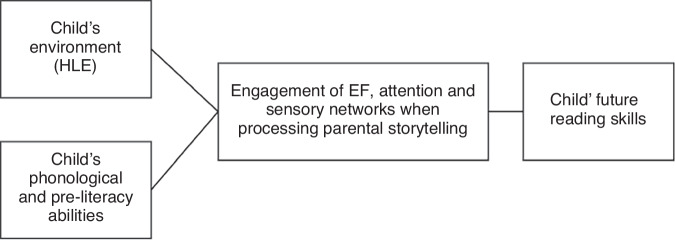
A summary of the relations between the study’s components.

While the child’s first exposure to literacy ideally is at home, it is important for children to be involved in reading activities early, in various environments and from additional trusted adults; all types of storytelling are beneficial for the growing child’s brain. Here, our results (see Fig. 3 for a schematic representation of the study’s components) support parental storytelling as a method of triggering neural networks supporting future reading abilities, specifically with parent involvement and its significant role in creating a most supportive neural environment optimal for reading fluency.

## Supplementary information

Supplementary information
