# Supplementary material for: Engagement of networks related to attention, executive function, and sensory processing during parental vs experimenter story-listening: an fMRI study
Source: Pediatr Res. 2025 Aug 4;99(3):1193–201. doi: 10.1038/s41390-025-04297-2 (PMC13021520; doi:10.1038/s41390-025-04297-2)

Supplemental materials

The following set of questionnaires and tests was administered in a separate room to assess participants' behavioral and cognitive abilities:

General verbal and nonverbal abilities: To ensure an average nonverbal ability, the Wechsler Preschool and Primary Scale of Intelligence's WPPSI matrix reasoning test for nonverbal intelligence was administered [22, 23] and verbal ability was determined using the expressive vocabulary test [22, 23]. Cronbach's alphas range from about .80 to .95, with composite score test-retest reliability from .86 to .91.

Executive Function: A version of the Behavior Rating Inventory of Executive Function (BRIEF-2) [24] asked parents to rate their child's executive functioning across 86 items assessing behaviors such as inhibition, shifting attention, emotional control, organization, and working memory, administered via paper or electronically, with three answer options for each item (frequency never, sometimes, always). The Global Executive Composite (GEC) score is the summary score derived from the indices Behavioral Regulation, Emotional Regulation, and Cognitive Regulation. Reliability coefficients (Cronbach's alpha) for the sub-indices range from .85 to .93.

Linguistic measures: Two tests from the Shatil Tests for Early Detection of Language Difficulties [25] were used to assess linguistic measures. The Naming task assesses semantic and verbal fluency by asking participants to quickly and accurately name five pictures of common items arranged in seven rows and

three columns, then five colors out loud arranged similarly (21 items per subtest, untimed); the 21-item form is adapted from a longer RAN subtest, which has a high test re-test reliability at .92. The Phoneme Awareness (Isolation) task asked participants to utter the sound made by the first letter of 16 one-syllable consonant-vowel-consonant pseudo-words spoken by the test administrator (what sound the word started with) [41]; alpha internal reliability is .92.

Pre-reading skills (Phonological Processing): Children were administered the digit-span subtest of the Comprehensive Test of Phonological Processing (CTOPP) [32], where they were asked to repeat a series of numbers read aloud by the test administrator in increasing length; internal consistency scores for the composites of CTOPP are .85 or greater.

Home literacy environment (HLE): The Stim Q2-Preschool questionnaire [26], or a shortened screener version transcribed in the lab [Educational Neuroimaging Group], was used to evaluate home literacy environment by asking about the amount and frequency of literary and play stimulation provided to the child within the home. The PRMSE reliability scores for the subsections within the StimQ2-Preschool (54 months) range from .476 to .807.

Reading Abilities: Reading measures at reading age included assessing fluency (accuracy and speed) during a contextual reading task from the Aleph-Taph battery[33] where participants were asked to read aloud a 99-word text with punctuation, Cronbach's alpha for the Word Reading Fluency task is .90.

Scatter plots for the correlation analyses

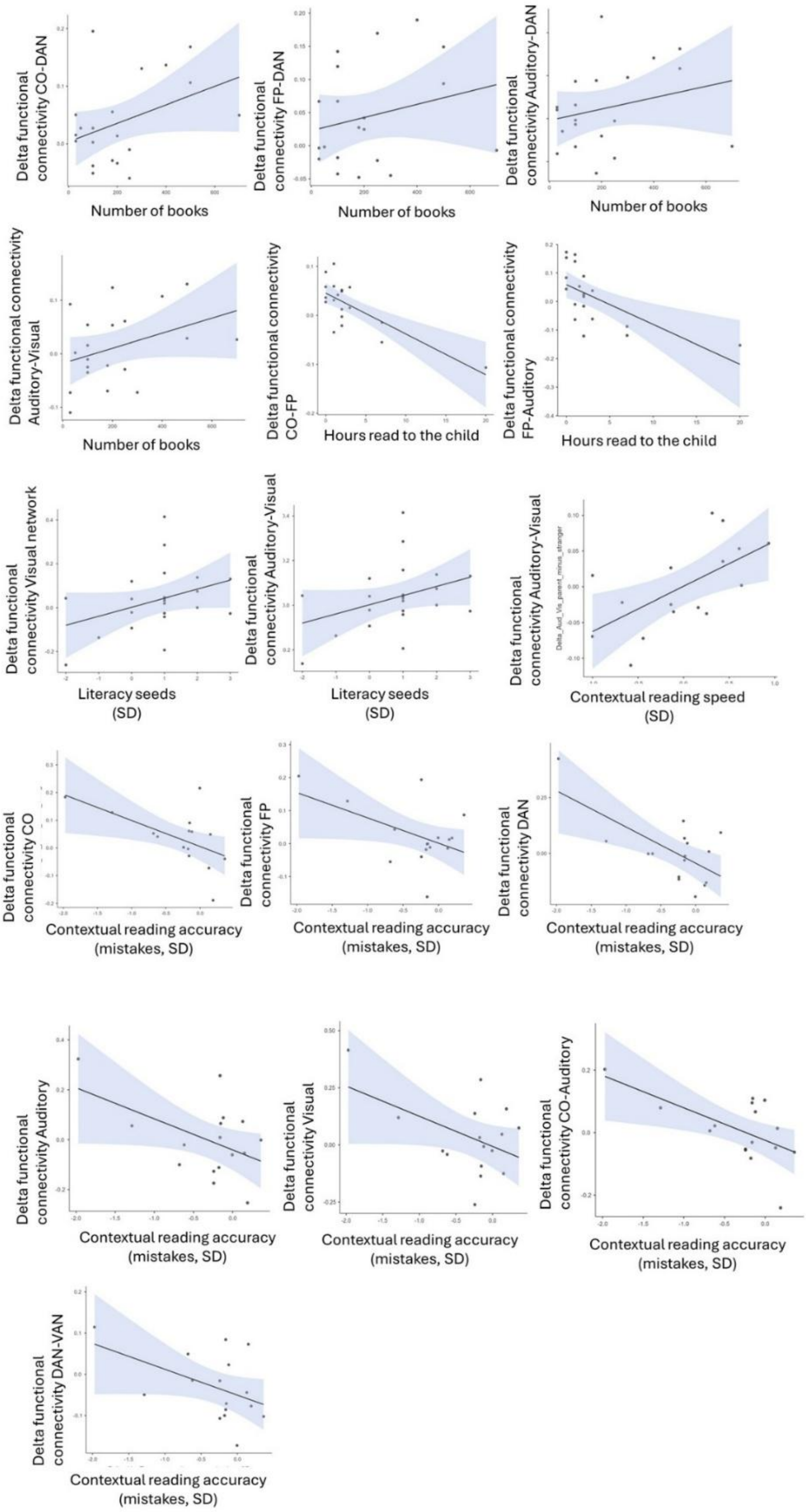

Supplement: Supplementary file 1 — Supplementary information [file 41390_2025_4297_MOESM1_ESM.pdf]
